# Pulsed Electromagnetic Field Inhibits Synovitis via Enhancing the Efferocytosis of Macrophages

**DOI:** 10.1155/2020/4307385

**Published:** 2020-05-26

**Authors:** Junjie Ouyang, Bin Zhang, Liang Kuang, Peng Yang, Xiaolan Du, Huabin Qi, Nan Su, Min Jin, Jing Yang, Yangli Xie, Qiaoyan Tan, Hangang Chen, Shuai Chen, Wanling Jiang, Mi Liu, Xiaoqing Luo, Mei He, Zhenhong Ni, Lin Chen

**Affiliations:** Department of Wound Repair and Rehabilitation Medicine, Center of Bone Metabolism and Repair, Laboratory for Prevention and Rehabilitation of Training Injuries, State Key Laboratory of Trauma, Burns and Combined Injury, Trauma Center, Research Institute of Surgery, Daping Hospital, Army Medical University (Third Military Medical University), Chongqing 400042, China

## Abstract

Synovitis plays an important role in the pathogenesis of arthritis, which is closely related to the joint swell and pain of patients. The purpose of this study was to investigate the anti-inflammatory effects of pulsed electromagnetic fields (PEMF) on synovitis and its underlying mechanisms. Destabilization of the medial meniscus (DMM) model and air pouch inflammation model were established to induce synovitis in C57BL/6 mice. The mice were then treated by PEMF (pulse waveform, 1.5 mT, 75 Hz, 10% duty cycle). The synovitis scores as well as the levels of IL-1*β* and TNF-*α* suggested that PEMF reduced the severity of synovitis in vivo. Moreover, the proportion of neutrophils in the synovial-like layer was decreased, while the proportion of macrophages increased after PEMF treatment. In addition, the phagocytosis of apoptotic neutrophils by macrophages (efferocytosis) was enhanced by PEMF. Furthermore, the data from western blot assay showed that the phosphorylation of P38 was inhibited by PEMF. In conclusion, our current data show that PEMF noninvasively exhibits the anti-inflammatory effect on synovitis via upregulation of the efferocytosis in macrophages, which may be involved in the phosphorylation of P38.

## 1. Introduction

The synovial membrane is a connective tissue membrane that is coated as the inner surface of the synovial joint capsule, which plays an important role in the maintenance of joint homeostasis [1]. Abnormal stimuli can cause synovitis, which is associated with a variety of joint diseases including rheumatoid arthritis (RA) [2] and osteoarthritis (OA) [3]. Synovitis greatly contributes to the pain in patients with arthritis. For RA, MRI-assessed synovitis is related to joint pain [4]. Moreover, surgical removal of synovium can effectively alleviate the pain of patients with RA [5–7]. Consistently, multiple clinical studies have also reported that synovitis is associated with pain in OA patients [1, 8, 9]. The pain intensity was accordingly changed with the degree of synovitis [10, 11]. In addition, synovitis plays an important role in the pathogenesis of arthritis [1–3, 12]. Targeting synovitis would be a potent strategy for the treatment of arthritis in the future.

PEMF is a noninvasive and safe treatment that commonly used in clinic to treat a variety of diseases/injuries including nonhealing fractures, postoperative pain, that edema, and osteoarthritis [13, 14]. Li et al. reported that electromagnetic fields are beneficial for relieving pain in patients with OA [15]. In addition, Gobbi et al. reported that PEMF treatment for symptomatic early knee OA patients can improve symptoms and knee function during a 1-year follow-up period [16]. Clinical studies by Bagnato et al. also showed that PEMF treatment can effectively reduce pain in patients with knee OA [17]. In addition to clinical studies, the therapeutic effects of PEMF have also been reported in animal models. Zhou et al. observed that PEMF can inhibit cartilage degeneration in a rat OA model induced by anterior cruciate ligament transection (ACLT) [18]. Ciombor et al. and Fini et al. have reported that PEMF can delay the development of lesions in the spontaneous OA model in guinea pigs [19, 20]. PEMF is reported not only to be useful in the treatment of animal models of OA but also effective in the treatment of animal models of RA. Kumar et al. and Selvam et al. have reported that PEMF can reduce edema volume of adjuvant induced arthritis (AIA) in rats [21, 22]. Considering the essential role of inflammation in the pathogenesis of OA and arthredema, these facts indicate the effectiveness of PEMF treatment in vivo against inflammation.

Previous studies reported that PEMF could inhibit the secretion of inflammatory factors including IL-1*β* and TNF-*α* in several types of cells [23–25], indicating that the PEMF has a certain anti-inflammatory effect. Murray et al. found that PEMF can reduce the release of lysosomal enzymes from rabbit synovial fibroblasts without affecting the release of collagenase and prostaglandin E2 [26]. De Mattei et al. reported that PEMF can reduce the release of prostaglandin E2 from bovine synovial fibroblasts, and this inhibition is achieved by upregulating adenosine receptor [27]. In addition, PEMF exerts anti-inflammatory effects by activating adenosine receptors in human OA synovial fibroblasts [28]. These data suggest that PEMF may influence the inflammatory response in synovium. We speculated that PEMF may have a regulatory effect on synovitis. However, the role and the detailed underlying mechanisms of the therapeutic effects of PEMF on synovitis are little known.

The purpose of this study was to investigate the anti-inflammatory effects of PEMF on synovitis and its underlying mechanisms. Our present study demonstrates that PEMF inhibited the synovitis in the surgery-induced posttraumatic synovitis model and in the air pouch model. PEMF inhibited the phosphorylation of P38, which may lead to the decrease of TNF-*α* and the increased efferocytosis in macrophages. The increased efferocytosis in macrophages may promote the resolution of inflammation and ultimately inhibit synovitis. Our research provides new insights into the underlying mechanisms of the anti-inflammatory effects of PEMF regarding the synovitis, which may be beneficial to the noninvasive treatment of arthritis in the future.

## 2. Materials and Methods

### 2.1. Electromagnetic Field Devices

The electromagnetic field device is customized by Sichuan Shangjian Chuangwei Medical Equipment Co., Ltd. It consists of four parts: the functional signal generator, power amplifiers, solenoid, and gauss meter. This electromagnetic field device can generate four waveforms, namely, sine wave, square wave, saw tooth wave, and pulse wave, with the intensity range of 0-10 mT and the frequency range of 0-100 Hz. The electromagnetic field parameters used in the present experiment were as follows: pulse waveform, 1.5 mT, 75 Hz, and 10% duty cycle [27, 29, 30].

### 2.2. Animal

Animal disposal method of this experiment is in line with the animal ethics standards. The research protocol has been approved by the Animal Ethics Review Committee of Daping Hospital. The male C57BL/6 (6-8 weeks old, 20 ± 1 g) mice were used for the experiment, and the DMM surgery was performed according to our previously reported method [31, 32]. The mice were randomly divided into 3 groups: (1) negative control (NC) group, (2) DMM surgery group, and (3) DMM+PEMF group. After DMM surgery, the DMM+PEMF group was treated with PEMF (2 h/d, 5 d/w). After two weeks, the mice were sacrificed by cervical dislocation, and the knee joints were fixed in 4% PFA for at least 24 hours. Air pouch model was established according to references [33]. The mice were randomly divided into 3 groups: (1) NC group, (2) lipopolysaccharide (LPS) treatment group, and (3) LPS+PEMF group. The mice in the NC group were injected with 1 ml saline after anesthesia, and the other two groups were injected with the same amount of LPS (Sigma, L4391) (50 *μ*g/ml, dissolve with saline). After waking up, the mice were treated with PEMF for 2 hours, followed by the rest for 1 hour, and then treated for another 2 hours. At the 8th hour, the mice were sacrificed, and the air pouches were washed with 1 ml saline. The lavage fluid was gathered and stored at -20°C. One small piece of air pouch skin was collected and fixed in 4% PFA for at least 24 hours.

### 2.3. Enzyme-Linked Immunosorbent Assay (ELISA)

The levels of IL-1*β* and TNF-*α* in the lavage fluid were evaluated using the Mouse IL-1*β* ELISA kit (Beyotime, PI301) and Mouse TNF-*α* ELISA kit (Beyotime, PT512). The measurement was carried out in accordance with the product manual.

### 2.4. Histological Analysis

The joint tissue was decalcified in 15% EDTA for 2 weeks. Then, all tissues were dehydrated and embedded with Frozen Section Medium (Thermo Scientific, 6502). The samples were sectioned with a freezing microtome (Leica CM3050 S Research Cryostat) and stained with hematoxylin and eosin. Synovitis of knee tissue was scored according to reference [34]. Briefly, the evaluation score for knee synovitis is based on five parts, including pannus, bone erosion, synovial hyperplasia severity, subsynovial inflammation, and synovial exudate. Pannus can be rated from zero to three points (0-3) based on severity (0: no pannus, 1: mild, 2: moderate, and 3: severe). Bone erosion can be rated 0-3 according to the loss range of cortical bone (0: no, 1: partial, 2: focal, and 3: widespread). Synovial hyperplasia severity can be rated 0-3 according to the degree of cell thick (0: 1, 1: 2~3, 2: 4~5, and 3: >6). Subsynovial inflammation can be rated 0-3 based on the degree of infiltration of inflammatory cells (0: no, 1: occasional scattered, 2: focal dense, and 3: widespread dense). Synovial exudate can be rated 0-1 based on the presence or absence of inflammatory cells or fibrin in synovial cavity (0: no, 1: yes). In addition, synovial-like layer cells of skin tissue were counted using ImageJ. A complete low-power field of view was selected for each mouse to calculate the cell number. At least 4 mice in each group were used for statistical calculations.

### 2.5. Immunofluorescence

Frozen sections were rewarmed in a 37°C incubator. After washing with PBS for 3 times, blocking was done with the QuickBlock™ Blocking Buffer for Immunol Staining (Beyotime, P0260). After removal of blocking buffer, diluted Ly-6G (BioLegend, 127636) primary antibody or F4/80 (Abcam, ab6640, 1 : 200) primary antibody with working solution were added and incubated overnight at 4°C. After washing 3 times with PBS, a working-solution diluted Goat Anti-Rat IgG (H+L) Cross-Adsorbed Secondary Antibody, Alexa Fluor 568 (Thermo Scientific, A-11077, 1: 800), was added and incubated at 37°C for 1 hour. DAPI dye solution were added and incubated at 37°C for 10 minutes as a counterstain of nucleus. Finally, after washing 3 times with PBS, 60% glycerol was placed on the slide. Fluorescence was observed with a confocal microscope (Carl Zeiss, LSM880NLO). The number of positive cells was counted manually. Three high-power fields in each sample were randomly selected to calculate the ratio between the number of fluorescent and DAPI positive cells. More than 3 mice in each group were used for statistical calculations.

### 2.6. Bone Marrow Neutrophils and Bone Marrow-Derived Macrophages (BMDM)

Bone marrow neutrophils were separated according to references [35]. Briefly, C57BL/6 mice were sacrificed, and the tibia and femur of the mice were removed. The bone marrow was washed out and dispersed into single cell suspension. Three milliliters of Histopaque-1119 (SIGMA, 11191), 3 ml of Histopaque-1077 (SIGMA, 10771), and the cell suspension were sequentially added into a 15 ml centrifuge tube to be centrifuged (700 g, 30 min, brake off) to have stratification. The cells between the 1119 and 1077 fluid layers were collected as polymorphonuclear neutrophil. After being washed with HBSS twice, neutrophils were labelled with the CFDA SE Cell Proliferation and Tracer Detection Kit (Beyotime, C0051) and then transferred to DMEM/HIGH GLUCOSE (HyClone, SH30022.01) complete medium to be cultured for 24 hours to induce apoptosis [36–38]. Similar to the above method, the isolated bone marrow cells were cultured in RPMI complete medium. One day later, suspension cells were collected and cultured for at least 6 days with 100 ng/ml macrophage colony-stimulating factor (M-CSF) (PeproTech, 315-02) to obtain BMDM. All cells were cultured at 37°C under 5% CO2 and 10% FBS (Gibco, 10099-141), and 1% Penicillin-Streptomycin Solution (HyClone, SV30010) was added.

### 2.7. Efferocytosis

RAW264.7 cells were cultured in a DMEM/HIGH glucose complete medium. When the cells density reached 90%, the cells were passaged at a dilution of 1 : 4. The RAW264.7 cells in a logarithmic growth phase were seeded into 6-well plates. After being treated with PEMF for 1 hour, the RAW264.7 cells were cocultured with CFDA-labelled apoptosis neutrophils for 4 hours. The cells were subsequently washed three times with PBS to remove away neutrophils that were not phagocytized. Attached RAW264.7 cells were digested and analyzed by flow cytometry [36].

### 2.8. Western Blots (WB)

The RAW264.7 cells and BMDM were seeded in 6-well plates. The cells were stimulated by LPS (Sigma, L8274) for 6 hours and then treated with PEMF for 30 minutes. After removing the supernatant, the cells were collected and subjected to cell lysate (Beyotime, P0013) for protein extraction. After being boiled for 10 minutes, 20 *μ*l of cell protein solution was separated by 12% SDS-PAGE gel and transferred to PVDF membrane. After blocking for 1 hour, the membrane was incubated with specific primary antibodies in 4°C overnight and washed with PBST 3 times. Finally, the membrane was incubated with anti-rabbit IgG or anti-mouse IgG at 37°C for 1.5 hours and washed 3 times with PBST. Immunoreactivity was visualized by chemiluminescence (ECL).

### 2.9. Reverse Transcription-Polymerase Chain Reaction (RT-PCR)

Total RNA extraction was performed by the TRIzol™ reagent (Thermo Scientific, 15596018), and RT-PCR was performed with the PrimeScript™ RT reagent Kit (Perfect Real Time) (Takara, RR037A). The operation was carried out according to the previous literature of our laboratory [32]. The oligonucleotide primer pairs used are as follows: IL-1*β* (forward: 5′-TGGACCTTCCAGGATGAGGACA-3′; reverse: 5′-GTTCATCTCGGAGCCTGTAGTG-3′); TNF-*α* (forward: 5′-CAGCCTCTTCTCCTTCCTGA-3′; reverse: 5′-CAGCTTGAGGGTTTGCTACA-3′); and Cyclophilin-A (forward: 5′-CGAGCTCTGAGCACTGGAGA-3′; reverse 5′-TGGCGTGTAAAGTCACCACC-3′). Cyclophilin-A was used as an internal control, and quantitative gene expression was normalized to Cyclophilin-A expression level.

### 2.10. Statistical

GraphPad Prism 7 was used for mapping and statistical analysis. Measurement data were expressed as mean ± SD. *t* test was employed for comparison between the two groups. One-way ANOVA followed by Tukey's post hoc test was used for comparison among three or four groups. *P* < 0.05 was considered statistically significant.

## 3. Results

### 3.1. PEMF Alleviates the Degree of Synovitis in the DMM Model

**Figure 1:**
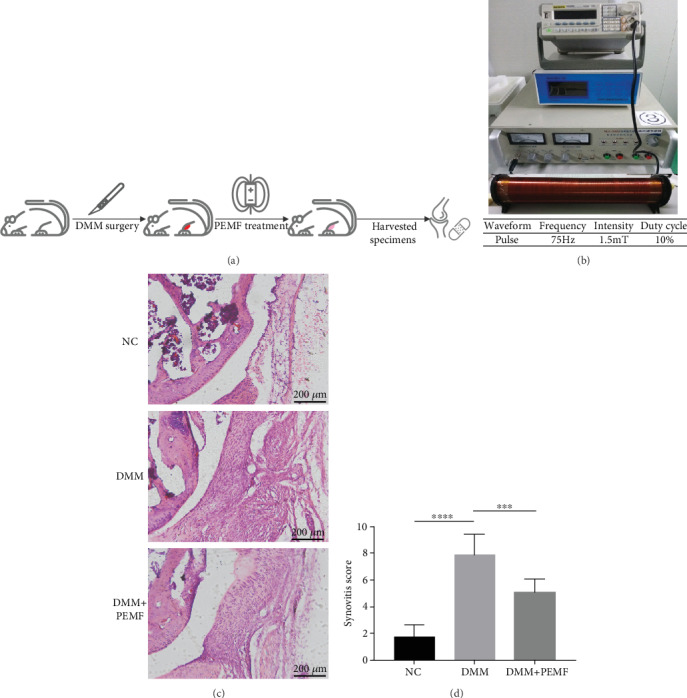
PEMF alleviates the degree of synovitis in the DMM model. (a) The procedure of mice treated with PEMF after DMM: 24 mice were randomly divided into three groups and used for the experiment. After DMM surgery, one group of mice was treated with PEMF. After two weeks, all mice were sacrificed and the medial compartment of the knee was histologically evaluated. (b) PEMF device and parameters used. (c) Histological evaluation: representative images of the medial compartment of the knee were showed. Scale bar: 200 *μ*m. (d) Synovitis score of each group was calculated. Statistical analysis was performed by one-way ANOVA, and multiple comparisons were performed using Tukey's test. Error bars represent the mean ± SD of eight independent experiments. ^∗∗∗^*P* < 0.001, ^∗∗∗∗^*P* < 0.0001.

As shown in Figure 1(a), the mice after DMM surgery were treated with PEMF for 2 weeks. The frequency of PEMF treatment was 2 hours a day, 5 days a week. All mice were sacrificed after two weeks of DMM surgery, and the knee joints were used for subsequent histological analysis. The PEMF device and parameters used are presented in Figure 1(b). The severity of synovitis was evaluated according to references based on the five aspects including pannus, bone erosion, synovial hyperplasia severity, subsynovial inflammation, and synovial exudate [34]. As shown in Figure 1(c), compared with the control group, pannus, synovial hyperplasia severity, and subsynovial inflammation was significantly aggravated in the DMM group, which were all alleviated by PEMF treatment, although still higher than that in basal levels. The quantified results of the synovitis score are shown in Figure 1(d). The severity of synovitis after DMM surgery was significantly worse than that of the control group, which were relieved after treatment with PEMF, but did not return to the basal levels. These data suggest that PEMF could reduce the degree of synovitis in the knee joint of DMM model.

### 3.2. PEMF Alleviates the Severity of Synovitis in the Air Pouch Model

**Figure 2:**
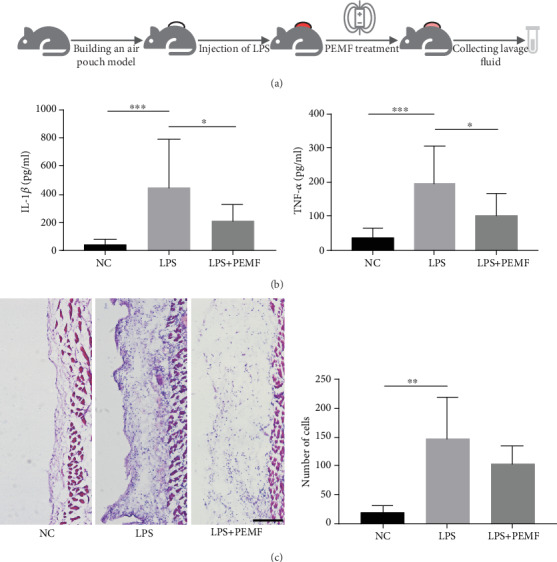
PEMF reduces the degree of synovitis in the air pouch model. (a) The procedure of mice treated with PEMF in air pouch model. After LPS was used to induce inflammation in the air pouches, the experimental group was treated with PEMF and the lavage fluid was collected. (b) The levels of IL-1*β* and TNF-*α* in the lavage fluid were measured by ELISA. Statistical analysis was performed by one-way ANOVA, and multiple comparisons were performed using Tukey's test. Error bars represent the mean ± SD of at least eight independent experiments. ^∗^*P* < 0.05, ^∗∗∗^*P* < 0.001. (c) A small piece of the skin from the air pouches was sampled for H.E. staining. H.E. staining showed no significant change in the number of nucleated cells after PEMF treatment. Statistical analysis was performed by one-way ANOVA, and multiple comparisons were performed using Tukey's test. Error bars represent the mean ± SD of four independent experiments. ^∗∗^*P* < 0.01. Scale bar: 100 *μ*m.

Next, we investigated the effect of PEMF on synovitis using the air pouch model. Following the induction of inflammation by LPS in the air pouches, the LPS+PEMF group was treated with PEMF and the lavage fluid from the air pouches was collected. The levels of IL-1*β* and TNF-*α* in the lavage fluid were measured by ELISA (Figure 2(a)). There were 10 mice in the NC group, 8 mice in the LPS group, and 11 mice in the LPS+PEMF group. As shown in Figure 2(b), the levels of IL-1*β* and TNF-*α* in the lavage fluid in the LPS group were significantly higher than that in the control group, which were reduced by PEMF treatment but still higher than that of the control group. These results indicate that PEMF could reduce the degree of inflammation in the air pouch model. To further investigate whether the reduced IL-1*β* and TNF-*α* were involved in the decreased inflammatory cell infiltration. We evaluated the number of inflammatory cells in the synovial-like layer by histological methods. As shown in Figure 2(c), the number of nucleated cells in the synovial-like layer of the LPS group was significantly increased compared with that of the NC group but was not significantly reduced after PEMF treatment. These data suggest that PEMF can inhibit the secretion of IL-1*β* and TNF-*α* in the air pouch model.

### 3.3. PEMF Treatment Changes the Proportion of Neutrophils and Macrophages in the Synovial-Like Layer of the Air Pouch Model

**Figure 3:**
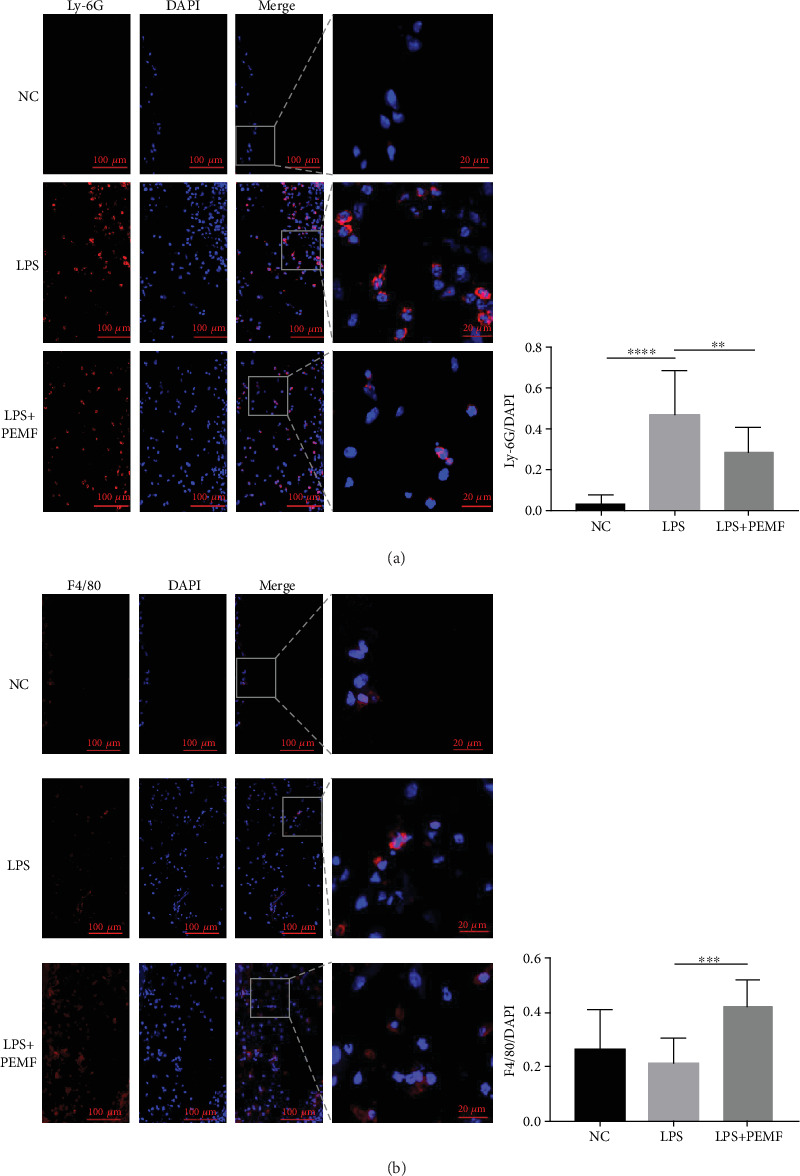
The proportion of inflammatory cells was changed after PEMF treatment. After LPS was used to induce inflammation in the air pouch, the experimental group was given PEMF treatment; a small piece of skin from the air pouch was sampled for immunofluorescent staining. (a) Immunofluorescence showed a decrease in Ly-6G-labelled neutrophils after PEMF treatment. Statistical analysis was performed by one-way ANOVA, and multiple comparisons were performed using Tukey's test. Error bars represent the mean ± SD of fifteen independent experiments. ^∗∗^*P* < 0.01, ^∗∗∗∗^*P* < 0.0001. (b) Immunofluorescence showed an increase in macrophages after PEMF treatment. F4/80-labelled macrophages. Statistical analysis was performed by one-way ANOVA, and multiple comparisons were performed using Tukey's test. Error bars represent the mean ± SD of at least eight independent experiments. ^∗∗∗^*P* < 0.001.

Neutrophils and macrophages are the main inflammatory cells in the synovial-like layer of the air pouch model [33, 39]. We further observed the proportion of neutrophils and macrophages in the synovial-like layer. Neutrophils and macrophages were distinguished by immunofluorescence via recognizing their specific markers. As shown in Figure 3(a), the neutrophils in the synovial-like layer of air pouches were labelled by Ly-6G antibody. After LPS treatment, the proportion of neutrophils in the synovial-like layer of air pouches was increased significantly, which was decreased after PEMF treatment but still significantly higher than the control group. As shown in Figure 3(b), macrophages were labelled with F4/80. Compared with the NC group, the proportion of macrophages did not change significantly after LPS treatment. This may be due to an increase in the number of macrophages and neutrophils after LPS treatment. After treatment with PEMF, the proportion of macrophage in the synovial-like layer of air pouches was statistically increased compared with the NC group and the LPS group. The results suggest that PEMF treatment changes the proportion of neutrophils and macrophages in the synovial-like layer of the air pouch model.

### 3.4. PEMF Treatment Enhances the Efferocytosis in Macrophages In Vitro

**Figure 4:**
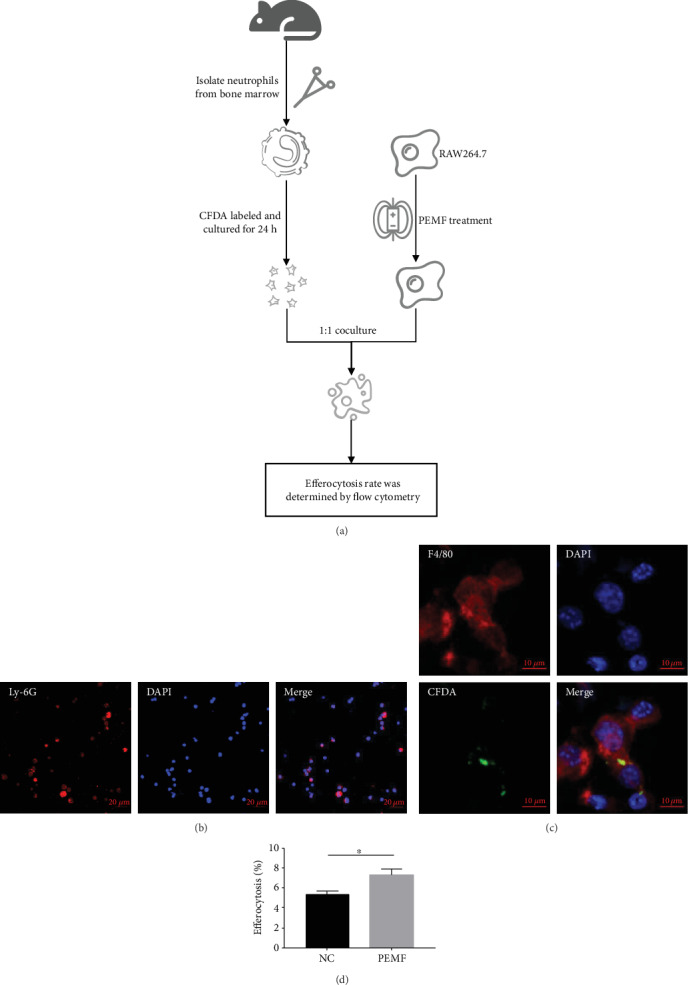
The efferocytosis in macrophages is increased by PEMF treatment. (a) The procedure of efferocytosis: neutrophils were isolated from the bone marrow and cultured for 24 hours after CFDA labelling to induce apoptosis. The RAW264.7 cells treated with PEMF for 1 hour were 1 : 1 cocultured with apoptotic neutrophils for 4 hours. The efferocytosis rate was measured by flow cytometry. (b) Neutrophil purity was detected by immunofluorescence after isolation of mouse bone marrow neutrophils. (c) A confocal microscope was used to observe the efferocytosis. (d) The percentage of efferocytosis was measured by flow cytometry. Statistical analysis was performed by *t* test. Error bars represent the mean ± SD of three independent experiments. ^∗^*P* < 0.05.

The activated macrophages can promote neutrophil clearance through efferocytosis [40]. Moreover, it has also been reported that PEMF can increase the phagocytic ability of macrophages [41, 42] and TNF-*α* can inhibit efferocytosis [38, 43]. Therefore, we deduced that PEMF may enhance efferocytosis by inhibiting the secretion of TNF-*α*, which leads to a change in the proportion of neutrophils and macrophages in the synovial-like layer. Therefore, the effect of PEMF on efferocytosis was further studied. The procedure for detecting the effect of PEMF on efferocytosis in macrophage is shown in Figure 4(a). Mouse primary neutrophils were isolated from the bone marrow and labelled with Ly-6G. As shown in Figure 4(b), the ratio of Ly-6G positive cells was 0.931 ± 0.039, which was roughly equivalent to the reported results [44]. The result indicates that the primary neutrophils of mice were successfully isolated/identified. It has been reported that most of the isolated primary neutrophils can undergo spontaneous apoptosis after 24 hours in vitro culture [36–38]. The isolated mouse primary neutrophils were labelled with CFDA and then cultured in DMEM-H complete medium for 24 hours to induce apoptosis. The RAW264.7 cells treated with PEMF for 1 hour were cocultured with CFDA-labelled apoptotic neutrophils for 4 hours at a ratio of 1 : 1. As shown in Figure 4(c), the colocalization of F4/80-positive RAW264.7 with CFDA-labelled neutrophil debris were observed under a confocal microscope, indicating that the RAW264.7 cells were capable of phagocytizing apoptotic neutrophils in this model. Furthermore, the efferocytosis of the RAW264.7 cells was increased after the PEMF treatment (Figure 4(d)). These data suggest that PEMF can enhance efferocytosis in the RAW264.7 cells.

### 3.5. PEMF Affects the Phosphorylation of P38 in Macrophages

**Figure 5:**
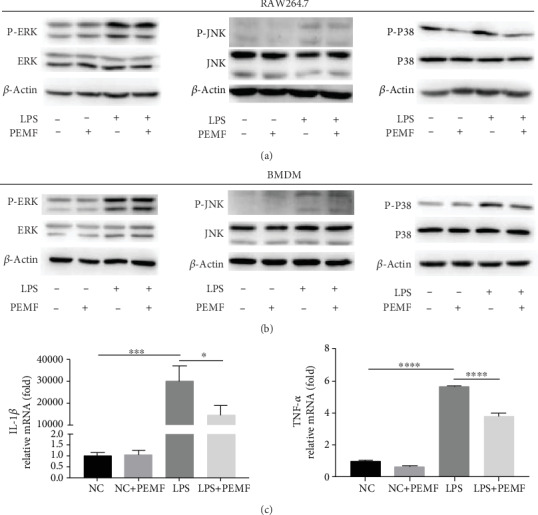
PEMF affects the phosphorylation of P38. The RAW264.7 cells (a) and BMDM cells (b) were stimulated with LPS for 6 hours and then treated with PEMF for 30 minutes. WB showed that there was no significant change in ERK and JNK, but P38 phosphorylation was weakened. (c) After extracting RNA from the RAW264.7 cells, the mRNA levels of IL-1*β* and TNF-*α* were detected by RT-PCR. Statistical analysis was performed by one-way ANOVA, and multiple comparisons were performed using Tukey's test. Error bars represent the mean ± SD of three independent experiments. ^∗^*P* < 0.05, ^∗∗∗^*P* < 0.001, ^∗∗∗∗^*P* < 0.0001.

Mitogen-activated protein kinase (MAPK), an important signalling transmitter in eukaryotic cells, participates in a wide variety of physiology/pathological processes including inflammation [45]. Then we investigate whether MAPK is involved in the effects of PEMF in macrophage. The RAW264.7 cells and BMDM cells were stimulated with LPS for 6 hours and then treated with PEMF for 30 minutes. WB was used to detect the effect of PEMF on related proteins level of MAPK signal. As shown in Figures 5(a) and 5(b), the PEMF had no significant effect on the total and phosphorylated level of JNK and ERK as well as the total protein of P38, but it inhibited the level of phosphorylated P38. Considering that P38 can affect the activation of transcription factors (such as AP1) to further change the expressions of inflammatory factors (such as IL-1*β* and TNF-*α*) [46], the expressions of IL-1*β* and TNF-*α* were detected in the RAW264.7 cells by RT-PCR. The RAW264.7 cells were stimulated with LPS for 6 hours and then treated with PEMF for 30 minutes. As shown in Figure 5(c), compared with the control group, the mRNA levels of IL-1*β* and TNF-*α* were significantly increased after LPS treatment, which was decreased after PEMF treatment but still higher than the control group. However, the group threated with PEMF alone did not exhibit significant changes compared to the control group. The results suggest that PEMF may inhibit the expressions of IL-1*β* and TNF-*α* via inhibiting P38 phosphorylation in LPS-treated macrophages.

## 4. Discussion

In this study, the DMM model and air pouch model were used to evaluate the effect of PEMF on synovitis. Although there are some differences between the synovitis models of the skin and knee, the air pouch model could be considered as an optional tool to investigate the pathological mechanisms of joint synovitis. After LPS injection, leukocytes represented by neutrophils and macrophages flow into the air pouches [33, 39], which is similar to the pathological process of synovitis. The air pouch model has been used to investigate some joint-related diseases including crystal arthritis [47], rheumatoid joints [48], and hydroxyapatite-related arthropathy [49]. Similarly, the DMM model is a posttraumatic OA model that induces OA by surgically destabilizing the medial meniscus [32, 50], which could be used to study the synovitis of the knee joint. Previous studies revealed that DMM induced inflammatory changes in synovium including pannus formation, synovial membrane hyperplasia, and subsynovial inflammatory cell infiltration [34, 51], which significantly increased after surgery and reached a peak at two weeks [34]. The synovitis induced by DMM could be alleviated by systemic blockade of IL-6 [52]. In this experiment, we used these two models to study the effects of PEMF on synovitis and underlying mechanisms. In addition, more research in other models such as AIA is needed to estimate the effect of PEMF on synovitis.

Macrophages play an important role in synovial inflammation [53, 54], and targeting synovial macrophages is considered as a potential strategy for regulating synovial inflammation. Some previous studies have found that PEMF can regulate the inflammatory response of macrophages. Ross et al. reported that PEMF can downregulate LPS-induced activation of NF*κ*B signal and decrease of TNF-*α* in RAW264.7 cells [55]. In addition, Akan et al. found that electromagnetic fields can inhibit the growth of *S. aureus* and increase the expression of HSP70 in macrophages [56]. Kubat et al. observed that PEMF treatment decreased the expression of proinflammatory cytokines and increased the level of anti-inflammatory cytokines in human monocytes [57]. These studies suggest that PEMF can exert anti-inflammatory effects by affecting the function of macrophages. In our present study, we firstly revealed that PEMF could enhance the macrophages-mediated efferocytosis. Efferocytosis can eliminate apoptotic cells to avoid the release of inflammatory factors from ruptured cells and can produce anti-inflammatory factors to inhibit inflammatory response [40], which greatly contributes to the resolution of inflammation. Therefore, our present study may provide new perspective to understand the anti-inflammatory mechanisms of PEMF based on macrophages-mediated efferocytosis.

P38 has been reported to play an important role in inflammatory response and considered as a possible anti-inflammatory molecular target [46, 58, 59]. Skepinone-L, a P38 inhibitor, can inhibit the severity of experimental arthritis in experimental arthritis model [60]. Inhibition of P38 by NJK14047 can downregulate the expression of various proinflammatory factors in LPS-treated BV2 microglia and can also reduce the activation of microglia in the brain of mice injected with LPS [61]. In addition, P38 was reported to be involved in the regulation of efferocytosis. Zhang et al. found that angiotensin II damages efferocytosis in advanced atherosclerosis, which is involved in P38 pathway [62]. Recently, D Gilroy et al. reported that inhibition of the elevated p38 MAPK could restore efferocytosis and enhance the inflammatory resolution in the elderly [63], indicating targeting P38 could be a potent strategy to modulate chronic inflammation via efferocytosis. In our study, we found that PEMF decreased the phosphorylation of P38 in macrophages, indicating the possible role of P38 in PEMF-mediated enhancement of efferocytosis. However, the detailed roles and mechanisms of P38 in this model still need further studies.

PEMF parameters are one of the important aspects affecting its therapeutic effect. However, the parameters of PEMF were variable in different experimental models. For in vitro models, Gómez-Ochoa et al. found that PEMF (15 min each time, treatment on days 7, 8, and 9 of culture) can reduce the secretion of IL-1*β* and TNF-*α* in human fibroblast-like cell [25]. Zou et al. treated the rat primary nucleus pulposus cells with PEMF (4 h/time, 2 times/day, total 7 days) and found that the secretion of IL-1*β* and TNF-*α* into the culture medium was reduced [23]. Fitzsimmons et al. found that a 30-minute PEF (pulsing electric field) can increase human chondrocyte proliferation [64]. For in vivo experiments, Sutbeyaz et al. found that PEMF (30 min/time, 2 times/day, total 3 weeks) treatment may improve the function, pain, fatigue, and overall condition of female patients with fibromyalgia [65]. Zhou et al. found that PEMF (40 min/day, 5 days/week for 12 weeks) treated ACLT rats had decreased cartilage degradation [18]. The PEMF parameters used in the present study are consistent with previous experiments (pulse waveform, 1.5 mT, 75 Hz, and 10% duty cycle) [27, 29, 30]. In addition, we also tried different PEMF time (2 h/4 h for in vivo model; 30 min/60 min for in vitro model) and selected the time used in this experiment. More researches are needed to estimate the effects of PEMF with different parameters so as to optimize the therapeutic effects of PEMF on synovitis and OA in the future.

## 5. Conclusions

**Figure 6:**
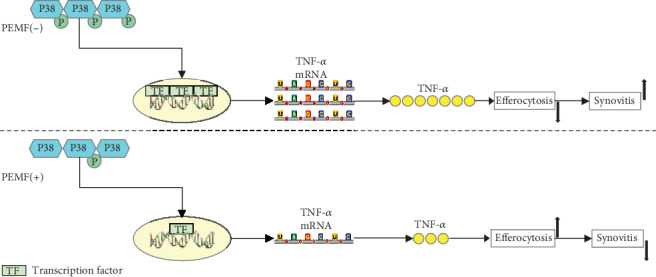
PEMF exerts anti-inflammatory effect through the P38/TNF-*α*/efferocytosis pathway. After treatment with PEMF, the phosphorylation of P38 is decreased, which decreases the transcription of TNF-*α* via affecting the downstream transcription factors. TNF-*α* is one of the cytokines that can inhibit efferocytosis and thus affect the resolution of inflammation. The PEMF reduces the production of TNF-*α* by inhibiting the phosphorylation of P38, thereby enhancing efferocytosis and promoting inflammation resolution.

In brief, our present study demonstrates that PEMF inhibits the synovitis in mouse DMM and air pouch model. PEMF inhibits the phosphorylation of P38, which may lead to the decrease of TNF-*α* and subsequently increased efferocytosis of neutrophils by macrophages. The increased efferocytosis in macrophages promotes the resolution of inflammation and ultimately inhibit synovitis (Figure 6). Our data provides new insights into the underlying mechanisms of the anti-inflammatory effects of PEMF regarding the synovitis, which may be beneficial to the noninvasive treatment of arthritis in the future.

## Data Availability

The detailed data used to support the findings of this study are available from the corresponding author upon request.
